# Hepatitis B immunity and response to booster vaccination in South African students following childhood immunisation

**DOI:** 10.4102/sajid.v41i1.803

**Published:** 2026-06-11

**Authors:** Dineo Monaheng, Pakiso Makhoahle, Sabeehah Vawda, Molefi D. Morobadi, Dewald Steyn, Lynette van der Merwe, Gina Joubert, Dominique Goedhals

**Affiliations:** 1 Department of Health Sciences, Faculty of Health and Environmental Sciences, Central University of Technology, Bloemfontein, South Africa; 2 Department of Virology, PathCare, Pretoria, South Africa; 3 Division of Virology, Faculty of Health Sciences, University of the Free State, Bloemfontein, South Africa; 4 Department of Virology, Ampath Laboratories, Pretoria, South Africa; 5 Department of Internal Medicine, Faculty of Health Sciences, University of the Free State, Bloemfontein, South Africa; 6 Division of Health Sciences Education, Faculty of Health Sciences, University of the Free State, Bloemfontein, South Africa; 7 Department of Biostatistics, Faculty of Health Sciences, University of the Free State, Bloemfontein, South Africa

**Keywords:** hepatitis B virus, healthcare students, anamnestic response, vaccination, immunity

## Abstract

**Background:**

Despite the introduction of hepatitis B virus (HBV) vaccination into the South African Expanded Programme on Immunization in 1995, HBV remains an important occupational risk to healthcare workers and students. Data regarding the duration and level of HBV immunity following childhood vaccination and response to booster vaccination are required to guide vaccination policies.

**Objectives:**

The study aimed to investigate pre- and post-vaccination immunity to hepatitis B in healthcare students born after implementation of infant immunisation.

**Method:**

Healthcare students were recruited from the Central University of Technology and the University of the Free State in Bloemfontein, South Africa. Hepatitis B surface antibody (HBsAb) levels were determined at baseline, month one and month seven, while booster vaccines were administered at 0 month, 1 month and 6 months. Hepatitis B total core antibody (HBcAb) was also tested at baseline. A total of 122 students were enrolled into the study from approximately 320 eligible students. Two participants did not return following the baseline visit, and one additional participant did not return for the final sampling visit.

**Results:**

At baseline, only 43% (*n* = 52/122) of participants had HBsAb levels which are considered to provide adequate protective immunity. Following a single booster, 92% (*n* = 111/120) of healthcare students had protective HBsAb levels, which increased to 100% (*n* = 119/119) following three vaccine doses. No significant differences were observed when HBsAb results were grouped by race or sex. Only one case of previously resolved HBV was identified.

**Conclusion:**

The study confirmed persistence of immunological memory in most healthcare students following childhood vaccination, as evidenced by anamnestic responses to a single dose of HBV vaccine.

**Contribution:**

Laboratory confirmation of adequate responses is essential to identify those requiring further vaccination and ensure protection in the event of HBV exposure.

## Introduction

Hepatitis B virus (HBV) is a vaccine-preventable viral infection, which can cause chronic hepatitis, cirrhosis and hepatocellular carcinoma. The World Health Organization (WHO) estimates that 254 million people were living with HBV globally in 2022, resulting in an estimated 1.1 million deaths per year.^1^

These carriers are more prevalent in developing and low-income countries.^2,3^ There are an estimated 65 million chronic hepatitis B surface antigen (HBsAg) carriers in Africa, including approximately 2.7 million in South Africa.^1^ South African studies have identified higher rates of HBV infection in males than females and in rural rather than urban areas.^4^

The WHO recommendations on childhood vaccination advise three doses of HBV vaccine for all children, with the first dose being administered as soon as possible but preferably within 24 h of birth.^5^ In South Africa, HBV vaccination was introduced into the Expanded Programme on Immunization (EPI) in April 1995 and is administered at 6 weeks, 10 weeks and 14 weeks of age, with an additional targeted birth dose for infants whose mothers tested HBsAg positive during pregnancy.^6^

Childhood vaccination is important not only to prevent vertical transmission but also to prevent horizontal infection, which is the principal route of transmission in Africa, including South Africa.^7,8^ Following the introduction of HBV vaccination into the South African EPI, there was a reduction in HBV prevalence in children, with HBsAg positivity rates dropping to 0.0% in children with unknown HIV status and 2.7% in children living with HIV.^6^ A recent systematic review reported protective anti-hepatitis B surface antibody (HBsAb) levels in 63.2% of individuals 10 years after childhood vaccination, declining by 6.6% per follow-up year.^9^ Both age at vaccination (lower prevalence of immunity in those vaccinated in infancy versus those vaccinated after 1 year or age) and the level of HBV endemicity (higher prevalence of immunity in highly endemic areas) affect rates. However, 90.34% of individuals showed an anamnestic response to a booster dose of vaccine, and the duration of functional immunity, which provides protection from infection, therefore likely persists despite waning antibody levels.^9^

Healthcare workers (HCWs) are considered at high risk of HBV exposure and have a four times greater probability of contracting HBV than the general population because of occupational exposure to blood and body fluids, as well as needlestick and sharps injuries.^10,11^ The risk of transmission following a percutaneous needlestick injury is 6% – 30% for HBV and approximately 0.3% for HIV.^12,13^ Up to 37% of HBV infections in HCWs result from occupational exposure via needle stick injuries.^14^ Studies in South Africa have established that 24% of needlestick injuries occur in primary care nurses and 69% among junior doctors per annum.^15,16^ Hepatitis B virus infection among HCWs remains high in South Africa, with HBV DNA positivity reported at 8.6%.^17^ Despite the recommendation from the South African National Department of Health that all healthcare students and in-service HCWs should receive three doses of HBV vaccine and have post-vaccination testing to confirm adequate antibody responses, vaccination coverage rates remain low among these groups.^18,19,20^ Coverage among healthcare students is impacted by institutional policies that often require students to pay for their own vaccination and post-vaccination testing. With many students requiring financial support, it becomes a challenge for these students to be vaccinated as required.^20^ In order to guide vaccination policies for healthcare students who have received childhood vaccination as part of the EPI, this study aimed to investigate HBV immunity in these young adults and the response to booster vaccination.

## Research methods and design

### Study population and study layout

A prospective longitudinal study was conducted at the Division of Virology, National Health Laboratory Service and University of the Free State (UFS) in Bloemfontein, South Africa. Participants were recruited during 2018 from approximately 160 first-year medical students from the Faculty of Health Sciences, UFS and approximately 160 first- and second-year Biomedical Technology students from the Faculty of Health and Environmental Sciences, Central University of Technology (CUT). Students were informed of the study through a letter and during in-person orientation sessions.

Students willing to participate were then invited to return for the baseline visit, where the informed consent process was completed. Written informed consent was obtained from all participants. Gatekeeper approval was also obtained from both academic institutions. For confidentiality, participants were allocated a study number, with all data deidentified prior to data analysis.

As childhood HBV vaccination was included in the South African EPI programme from 1995, students were eligible to participate in the study if born in South Africa during or after 1995. Students were excluded from the study if they reported receiving HBV vaccination prior to the study at any time following childhood vaccination. Most students not participating in the study had elected not to do so and were not contacted to ascertain the underlying reasons for refusing participation.

A total of 122 participants were enrolled in the study, from the approximately 320 eligible students.

Two participants did not return following the baseline visit and therefore could not be included for subsequent testing, and one additional participant did not return for the final sampling at month seven. At the baseline visit, participants completed a questionnaire including demographic information (age, sex, race), HBV vaccination history and clinical history, including previous jaundice, previous confirmed HBV infection and history of other liver disease. Three doses of HBV vaccine (Heberbiovac-HB, Centre for Genetic Engineering and Biotechnology, Havana, Cuba) were administered at 0 month, 1 month and 6 months. Heberbiovac-HB is a recombinant HBsAg vaccine administered intramuscularly at a dose of 1.0 mL (20 µg HBsAg). Blood samples were collected at baseline, 1 month after the first vaccine dose (prior to receiving the second dose) and 1 month after the final dose (month seven). At each time point, up to 9 mL of venous blood was collected using BD Vacutainer^®^ Serum Blood Collection Tubes (Becton Dickinson, New Jersey, United States [US]). The clotted blood samples from each time point were centrifuged at 3000 revolutions per minute (rpm) for 10 min after which the serum was aliquoted and stored at –80 °C for later testing.

### Laboratory testing

Quantitative determination of HBsAb was performed on samples from each time point using the Liaison^®^ Anti-HBs II assay (DiaSorin, Saluggia, Italy), according to the manufacturer’s instructions. Hepatitis B surface antibody results were classified as negative (< 9.00 mIU/mL), equivocal (9.00 mIU/mL – 10.99 mIU/mL) or positive (≥ 11.00 mIU/mL) according to the assay manufacturer’s recommendations. According to the Centre for Disease Control (CDC), a level above 10 mIU/mL is considered protective.^21^ For the purposes of the study, only samples with a value ≥ 11 mIU/mL were considered positive and thus protective, in order to factor in the manufacturer’s equivocal range and prevent misclassification.

Baseline testing also included hepatitis B total core antibody (HBcAb) using the Liaison^®^ Anti-HBc assay (DiaSorin, Saluggia, Italy). Hepatitis B total core antibody results were classified as negative (≥ 1.100), equivocal (0.900–1.099) or positive (< 0.900) according to the index values recommended by the assay manufacturer.

### Statistical analysis

Deidentified data were captured electronically using Microsoft Excel. Laboratory and demographic results were summarised by frequencies and percentages (categorical variables). At each time point, the distribution of HBsAb levels was compared between the sexes and between race groups, using Fisher’s exact tests because of sparse cells. The statistical analysis was performed using SAS software, version 9.4 (SAS Institute Inc., Cary, NC, United States).

### Ethical considerations

Ethical clearance to conduct this study was obtained from the University of the Free State Health Sciences Research Ethics Committee (No. HSREC 79/2017 [UFS-HSD2017/0788 and UFS-HSD2018/0783/3107]).

## Results

### Demographic characteristics

The 122 students recruited into the study included 67 participants from CUT and 55 from UFS. The participants were all aged between 18 and 22 years, with the majority being female (*n* = 74/122, 61%) (Table 1). Most participants were African (*n* = 92.75%), followed by Caucasian (*n* = 20.17%).

**TABLE 1:** Participants’ demographic characteristics (*N* = 122).

Variable	*n*	%
**Institution**		
CUT	67	55
UFS	55	45
**Age (years)**		
18	42	34
19	34	28
20	21	17
21	22	18
22	3	3
**Sex**		
Male	48	39
Female	74	61
**Race**		
African people	92	75
Asian people	9	7
Caucasian people	20	17
Mixed race people	1	1

CUT, Central University of Technology; UFS, University of the Free State.

### Vaccination history and clinical data

Only one participant could confirm HBV vaccination during childhood and provided immunisation records confirming receipt of three doses of HBV vaccine. The remaining participants were unaware of their childhood HBV vaccination status. Participants were questioned regarding previous HBV infection or other liver diseases. A total of 113 students (93%) confirmed no history of previous HBV infection, while 9 (7%) were uncertain of previous HBV infection. Regarding a history of jaundice, 2 (2%) reported a history of neonatal jaundice, 116 (95%) had no previous history of jaundice, 1 (1%) was not certain and 3 (2%) were previously diagnosed with jaundice without a specified cause. For other liver conditions, 117 (96%) confirmed no history of other liver disease, while 5 (4%) were uncertain.

### Hepatitis B serology

Prior to receiving any booster vaccine doses, 52% of participants tested HBsAb negative (*n* = 64), 43% tested positive (*n* = 52) and 5% were equivocal (*n* = 6) (Table 2). Two equivocal samples had HBsAb values between 10.00 mIU/mL and 10.99 mIU/mL and would therefore have been considered protective according to the CDC classification, while the remaining four samples ranged between 9.00 mIU/mL and 9.99 mIU/mL. Two participants did not return for the second visit and therefore only had baseline information available. One month after the first booster dose of HBV vaccine, 8% of participants (*n* = 9/120) remained HBsAb negative, with the remainder having titres above 11 mIU/mL.

**TABLE 2:** Pre- and post-vaccination hepatitis B surface antibody levels of South African healthcare students.

HBsAb	Baseline (*N* = 122)		Month one (*N* = 120)		Month seven (*N* = 119)	
	*n*	%	*n*	%	*n*	%
Negative (< 9.00 mIU/mL)	64	52	9	8	0	0
Equivocal (9.00 mIU/mL – 10.99 mIU/mL)	6	5	0	0	0	0
Positive (≥ 11.00 mIU/mL)	52	43	111	92	119	100

HBsAb, hepatitis B surface antibody.

One participant completed the three-dose vaccination series but did not return for the final blood sample collection. One month after the third vaccination, all participants had HBsAb levels > 11 mIU/mL (*n* = 119), with 115 (96%) participants at > 1000 mIU/mL and one participant each with titres of 969 mIU/mL, 476 mIU/mL, 436 mIU/mL and 11 mIU/mL.

At each time point, the distribution of HBsAb levels was compared between the sexes and between race groups. No statistically significant differences were found at any of the time points (see Table 3).

**TABLE 3:** Pre- and post-vaccination hepatitis B surface antibody levels and hepatitis B total core antibody of South African healthcare students according to sex and race.

Variable	Baseline HBsAb (*N* = 122)						*p*-value	Baseline HBcAb (*N* = 122)				Month one HBsAb (*N* = 120)						*p*-value	Month seven HBsAb (*N* = 119)						*p*-value
	Negative		Equivocal		Positive			Negative		Positive		Negative		Equivocal		Positive			Negative		Equivocal		Positive		
	*n*	%	*n*	%	*n*	%	*n*	%	*n*	%	*n*	%	*n*	%	*n*	%	*n*	%	*n*	%	*n*	%
**Sex**	-	-	-	-	-	-	0.862	-	-	-	-	-	-	-	-	-	-	1.000	-	-	-	-	-	-	0.561
Female	39	53	3	4	32	43	-	74	100	0	0	6	8	0	0	67	92	-	0	0	0	0	73	100	-
Male	25	52	3	6	20	42	-	47	98	1	2	3	6	0	0	44	94	-	0	0	0	0	46	100	-
**Race**	-	-	-	-	-	-	0.229	-	-	-	-	-	-	-	-	-	-	0.483	-	-	-	-	-	-	0.575
African people	53	58	5	5	34	37	-	92	99	1	1	9	10	0	0	82	90	-	0	0	0	0	90	100	-
Asian people	3	33	0	0	6	67	-	9	100	0	0	0	0	0	0	9	100	-	0	0	0	0	9	100	-
Caucasian people	7	35	1	5	12	60	-	20	100	0	0	0	0	0	0	19	100	-	0	0	0	0	19	100	-
Mixed race people	1	100	0	0	0	0	-	1	100	0	0	0	0	0	0	1	100	-	0	0	0	0	1	100	-

HBsAb, hepatitis B surface antibody; HBcAb, hepatitis B total core antibody.

Hepatitis B total core antibody testing was performed on all participants at baseline to identify current or previous HBV infection.

Only one participant, an African male, tested HBcAb positive (1%), while the remaining 121 (99%) tested negative (Table 3). The single participant who tested HBcAb positive also tested HBsAb-positive pre-vaccination with a titre of 418 mIU/mL, which increased to > 1000 mIU/mL following the first booster dose of HBV vaccine. The presence of both HBcAb and HBsAb indicates previous resolved HBV infection.

## Discussion

While both international and South African National Department of Health guidelines recommend a course of three doses of HBV vaccine for HCW, vaccination policies for healthcare students in South Africa are governed by the individual academic institutions and are poorly standardised.^20^ Policies surrounding vaccination of healthcare students are further complicated by the need to consider the changing vaccination profiles of new students, most of whom will now have been born after the implementation of universal HBV vaccination in South Africa. It is therefore necessary to establish how frequently incoming students who were born after the introduction of childhood HBV vaccination have protective HBsAb levels prior to booster vaccination and whether a single booster is sufficient to stimulate protective levels in the remainder. To this end, 122 healthcare students were recruited from two South African academic institutions in central South Africa. Only one participant provided childhood immunisation records to confirm receipt of three doses of HBV vaccine during routine EPI vaccinations.

The remainder were unaware of their vaccination status and without access to their childhood immunisation records. This indicates the need for exploring alternative methods of documenting immunisation interventions to improve record keeping and provide better continuity of care.

Prior to receiving the first booster vaccine dose, most participants had negative (52%) HBsAb results below the level considered reliably protective against HBV infection. These data support available South African data where inadequate HBsAb levels were noted in 56% of healthcare students in Gauteng prior to boosting.^22^ This may be because of waning immunity as a result of the time which had elapsed since childhood vaccination, non-response to childhood vaccination or missed or incomplete childhood vaccination.

Confirmation of antibody responses through laboratory testing is not included in the EPI in South Africa, and it is therefore possible that some of the participants had inadequate responses to their initial vaccinations. Although HBV vaccines are highly immunogenic and effective at preventing HBV transmission, HBV vaccination induces protective antibody levels in 85% – 93% of South African infants, with slightly lower response rates in those living with HIV (78%).^6^ Waning immunity likely accounts for most cases with inadequate HBsAb levels, which was supported by the rapid HBsAb production following exposure to a booster HBV vaccine dose. This anamnestic response on re-exposure to the antigen supports immunological memory induced by the initial vaccine course and is the mechanism by which individuals can remain protected even when antibody levels have waned.^9^ One month following the first booster dose, only 8% of participants remained HBsAb negative. The absence of an adequate antibody response in the 8% of students who remained HBsAb negative following the first vaccination indicates inadequate memory responses to prevent infection in the event of an exposure, likely because of absent or incomplete infant vaccination or non-response to the infant vaccination.

Following the third booster, all participants had HBsAb levels above 11 mIU/mL, and only one individual would be considered a low responder (10 mIU/mL – 100 mIU/mL). The rest of the students were high responders, defined as HBsAb levels above 100 mIU/mL.^23^

No non-responders were identified among this group; however, non-response to HBV vaccination is known to occur in 5% – 10% of individuals vaccinated against HBV and is associated with various factors including age, sex, obesity, smoking and immunosuppression.^23^ A previous study among South African healthcare students found non-response to three doses of vaccine in 7% (*n* = 5/71) of their study participants.^22^

This emphasises the importance of post-vaccination testing in healthcare students to confirm immunity prior to possible exposure in a clinical environment.

No statistically significant differences were found at any of the three time points when HBsAb results were grouped by sex or race. Race has previously been identified as having a significant association with pre-vaccination HBsAb levels in South African healthcare students, with higher levels among Indian than black students.^22^ While this was not substantiated by our data, the small number of participants in certain subgroups may have contributed to this lack of statistical significance.

Prior to the introduction of HBV vaccination in South Africa, the prevalence of HBsAg was 9.6% among South Africans adults, with 76% showing evidence of previous exposure as indicated by having one or more serological markers for HBV.^7^ Public sector laboratory data from South Africa showed a decreasing trend in HBsAg positivity rates between 2015 and 2019 and also in incidence rates of acute HBV infection, likely because of the efficacy of childhood vaccination in preventing HBV transmission.^24^ Similar trends of decreasing seropositivity have been observed in numerous other countries following the introduction of HBV vaccination.^25,26,27^ In this study, serological evidence of previous resolved infection was identified in a single participant who tested positive for both HBsAb and HBcAb at baseline, thus a positivity rate of 1%, supporting the efficacy of the childhood vaccination programme.

The study’s findings must be seen in the light of its limitations. These include selection bias that may have been introduced because of the limited sample size, with 122 of approximately 320 eligible students agreeing to participate in the study. While the reasons for non-participation were not elucidated, it is possible that there were differences between students who chose to participate and those who declined. Possible sampling bias may also limit the generalisability of the findings by the inclusion of students from only two institutions from a single province, as well as the predominance of females (61%). A study performed among healthcare students at the University of the Witwatersrand showed similar gender and race distribution of participating students, with a predominance of black (80%) and female students (70%).^22^

The unavailability of vaccination records is also a limitation of this study as participants may not have been vaccinated as infants or may have received subsequent doses that they were unaware of. It is also possible that some of the participants may not have received one or more doses of the childhood vaccination schedule at 6 weeks, 10 weeks and 14 weeks of age because of incomplete vaccination coverage, which can occur in the EPI programme. While the participants were all born after the implementation of HBV vaccination, the available WHO estimates for national immunisation coverage for receipt of the third dose of HBV vaccine in South Africa from 1997 to 2000 ranged from 74% to 88%.^28^ This variability in prior vaccination may have impacted the study’s findings regarding baseline immunity and the attribution of baseline and probable anamnestic immune responses to infant vaccination.

The findings highlight the need for better documentation of vaccination history, particularly given the incomplete coverage, which is well documented in the South African EPI programme. This could be addressed by implementing a national electronic immunisation registry of both childhood and adult vaccinations to improve long-term record retention. In addition, standardised vaccination and immunity testing policies for tertiary academic institutions would aid in ensuring that all healthcare students are adequately protected from HBV prior to exposure in a clinical setting. One approach would be to perform baseline HBsAb testing followed by one or more booster doses before retesting to identify those without adequate antibody levels. This is more cost effective than a post-exposure management approach.^21^ As most current healthcare students will have received HBV vaccine as infants, another approach is to test HBsAb levels following a single dose of vaccine, with administration of additional doses where needed.^20^

Given the low cost of recombinant HBV vaccines, it has also been suggested that administration of three doses of vaccine prior to determining HBsAb levels may be more cost effective.^20^ As per international guidelines, post-vaccination serological testing is essential in such programmes to confirm adequate immunity and to allow education and appropriate management of non-responders.^5,21^ This would also simplify long-term occupational health policies in the healthcare setting as further boosters are not required in immunocompetent individuals who have a documented response to HBV vaccination.^5,29^ Cost efficacy studies would assist in confirming which vaccination and testing algorithm would be most cost efficient in the South African setting and allow assessment of the feasibility of implementation of institutionally funded programmes, where not already in place. Institutional funding would improve coverage among healthcare students, particularly those requiring financial assistance and from historically disadvantaged universities.^20^ Similar institutional policies are also required for various other vaccine-preventable infectious diseases to which healthcare students may be occupationally exposed.

## Conclusion

This study confirmed the persistence of long-term immunological memory in healthcare students following vaccination as infants, with 92% of participants having evidence of protective levels of HBsAb following a single booster dose. It also highlights the importance of routine testing for HBsAb levels following vaccination in high-risk groups to confirm adequate protection in the event of occupational HBV exposure. This is critical to identify individuals who require three doses of HBV vaccine because of an inadequate response to a single booster and identify non-responders following the full course which, although not seen in this study, is a well-established phenomenon.

## References

[1] World Health Organization (WHO). Global Hepatitis Report 2024: Action for access in low- and middle-income countries [homepage on the Internet]. 2024 [cited 2025 Oct 08]. Available from https://www.who.int/publications/i/item/9789240091672#:~:text=Download%20(9%20MB)-,Overview,187%20countries%20across%20the%20world

[2] GBD 2019 Hepatitis B Collaborators. Global, regional, and national burden of hepatitis B, 1990–2019: A systematic analysis for the Global Burden of Disease Study 2019. Lancet Gastroenterol Hepatol. 2022;7(9):796–829. 10.1016/S2468-1253(22)00124-8 35738290 PMC9349325

[3] Yuen MF, Chen DS, Dusheiko GM, et al. Hepatitis B virus infection. Nat Rev Dis Primers. 2018;4:18035. 10.1038/nrdp.2018.35 29877316

[4] Kew MC. Hepatitis B virus infection: the burden of disease in South Africa. S Afr J Epi Inf. 2008;23(1):4–8. 10.1080/10158782.2008.11441293

[5] World Health Organization (WHO). Hepatitis B vaccines: WHO position paper – July 2017. Wkly Epidemiol Rec. [serial online]. 2017 [cited 2025 Oct 08];92(27):369–392. Available from: https://www.who.int/publications/i/item/who-wer922728685564

[6] Burnett RJ, Kramvis A, Dochez C, Meheus A. An update after 16 years of hepatitis B vaccination in South Africa. Vaccine. 2012;30(Suppl 3):C45–C51. 10.1016/j.vaccine.2012.02.021 22939021

[7] Kiire CF. The epidemiology and prophylaxis of hepatitis B in sub-Saharan Africa: A view from tropical and subtropical Africa. Gut. 1996;38(Suppl 2):S5–S12. 10.1136/gut.38.Suppl_2.S5 8786055 PMC1398049

[8] Nelson NP, Easterbrook PJ, McMahon BJ. Epidemiology of hepatitis B virus infection and impact of vaccination on disease. Clin Liver Dis. 2016;20(4):607–628. 10.1016/j.cld.2016.06.006 27742003 PMC5582972

[9] Ramrakhiani H, Le MH, Kam L, et al. Long-term immunity and anamnestic response following hepatitis B vaccination: A systematic review and meta-analysis. J Viral Hep. 2025;32(2):e70003. 10.1111/jvh.70003 39831733

[10] Lamberti M, De Rosa A, Garzillo EM, et al. Vaccination against hepatitis b virus: Are Italian medical students sufficiently protected after the public vaccination programme? J Occup Med Toxicol. 2015;10:41. 10.1186/s12995-015-0083-4 26539242 PMC4632277

[11] Di Giuseppe G, Nobile CG, Marinelli P, Angelillo IF. A survey of knowledge, attitudes, and behavior of Italian dentists toward immunization. Vaccine. 2007;25(9):1669–1675. 10.1016/j.vaccine.2006.10.056 17129642

[12] Beltrami EM, Williams IT, Shapiro CN, Chamberland ME. Risk and management of blood-borne infections in health care workers. Clin Micro Rev. 2000;13(3):385–407. 10.1128/CMR.13.3.385 10885983 PMC88939

[13] Baggaley RF, Boily MC, White RG, Alary M. Risk of HIV-1 transmission for parenteral exposure and blood transfusion: A systematic review and meta-analysis. AIDS. 2006;20(6):805–812. 10.1097/01.aids.0000218543.46963.6d 16549963

[14] Prüss-Ustün A, Rapiti E, Hutin Y. Estimation of the global burden of disease attributable to contaminated sharps injuries among health-care workers. Am J Ind Med. 2005;48(6):482–490. 10.1002/ajim.20230 16299710

[15] Karstaedt AS, Pantanowitz L. Occupational exposure of interns to blood in an area of high HIV seroprevalence. S Afr Med J. 2001;91(1):57–61. 11236300

[16] Kruger WH, Joubert G, Jimoh SO. Needlestick injuries among nurses in a regional hospital in South Africa. Occup Health South Afr [serial online]. 2012 [cited 2025 Oct 08];18(3):4–10. Available from: https://www.occhealth.co.za/_assets/articles/218/1351.pdf

[17] Sondlane TH, Mawela L, Razwiedani LL, et al. High prevalence of active and occult hepatitis B virus infections in healthcare workers from two provinces of South Africa. Vaccine. 2016;34(33):3835–3839. 10.1016/j.vaccine.2016.05.040 27265453

[18] Vardas E, Ross MH, Sharp G, McAnerney J, Sim J. Viral hepatitis in South African healthcare workers at increased risk of occupational exposure to blood-borne viruses. J Hosp Infect. 2002;50(1):6–12. 10.1053/jhin.2001.1143 11825045

[19] Mosendane T, Kew MC, Osih R, Mahomed A. Nurses at risk for occupationally acquired blood-borne virus infection at a South African academic hospital. S Afr Med J. 2012;102(3):153–156. 10.7196/SAMJ.4563 22380910

[20] Burnett RJ, Dramowski A, Amponsah-Dacosta E, Meyer JC. Increasing hepatitis B vaccination coverage of healthcare workers – Global lessons for South Africa. Curr Opin Immunol. 2021;71:6–12. 10.1016/j.coi.2021.03.010 33819774

[21] Schillie S, Murphy TV, Sawyer M, et al. CDC guidance for evaluating health-care personnel for hepatitis B virus protection and for administering postexposure management. MMWR Recomm Rep. 2013;62(10):1–19. 24352112

[22] Makan N, Song E, Kinge CW, Kramvis A. Hepatitis B virus immunity prior to and after administration of a ‘booster’ dose of vaccine among health-care students at a South African university. Vaccine X. 2023;14:100284. 10.1016/j.jvacx.2023.100284 37063305 PMC10090248

[23] Bello N, Hudu SA, Alshrari AS, Imam MU, Jimoh AO. Overview of hepatitis B vaccine non-response and associated B cell amnesia: A scoping review. Pathogens. 2024;13(7):554. 10.3390/pathogens13070554 39057781 PMC11279426

[24] Moonsamy S, Suchard M, Pillay P, Prabdial-Sing N. Prevalence and incidence rates of laboratory-confirmed hepatitis B infection in South Africa, 2015 to 2019. BMC Public Health. 2022;22(1):29. 10.1186/s12889-021-12391-3 34991533 PMC8739689

[25] Liang X, Bi S, Yang W, et al. Epidemiological serosurvey of hepatitis B in China-declining HBV prevalence due to hepatitis B vaccination. Vaccine. 2009;27(47):6550–6557. 10.1016/j.vaccine.2009.08.048 19729084

[26] Ott JJ, Stevens GA, Groeger J, Wiersma ST. Global epidemiology of hepatitis B virus infection: New estimates of age-specific HBsAg seroprevalence and endemicity. Vaccine. 2012;30(12):2212–2219. 10.1016/j.vaccine.2011.12.116 22273662

[27] Spearman CW, Afihene M, Ally R, et al. Hepatitis B in sub-Saharan Africa: Strategies to achieve the 2030 elimination targets. Lancet Gastroenterol Hepatol. 2017;2(12):30295–30299. 10.1016/S2468-1253(17)30295-9 29132759

[28] Franҫois G, Dochez C, Mphahlele MJ, Burnett R, Van Hal G, Meheus A. Hepatitis B vaccination in Africa: Mission accomplished? South Afr J Epidemiol Infect. 2008;23(1):24–28. 10.1080/10158782.2008.11441296

[29] Centers for Disease Control and Prevention (CDC). Responding to HBV exposures in health care settings [homepage on the Internet]. c2024 [updated 2024 May 16; cited 2026 Apr 14]. Available from: https://www.cdc.gov/hepatitis-b/hcp/infection-control/index.html

